# Using the spring constant method to analyze arterial elasticity in type 2 diabetic patients

**DOI:** 10.1186/1475-2840-11-39

**Published:** 2012-04-25

**Authors:** Ching-Chuan Wei, Shu-Wen Huang, Cho-Tsan Bau

**Affiliations:** 1 Department of Information and Communication Engineering, Chaoyang University of Technology, Taichung, Taiwan, (R.O.C; 2 Graduate Institute of Informatics, Chaoyang University of Technology and Taichung Hospital, Department of Health, Executive Yuan, Taiwan, (R.O.C; 3 Taichung Hospital, Department of Health, Executive Yuan, Taiwan, (R.O.C; 4 No. 6, 591 Lane, Yonglong Rd, Dali District, Taichung City, Taiwan, 412, (R.O.C

**Keywords:** Spring constant, Arterial elasticity, Type 2 diabetes, Carotid-femoral pulse wave velocity

## Abstract

**Background:**

This study tests the validity of a newly-proposed spring constant method to analyze arterial elasticity in type 2 diabetic patients.

**Methods:**

The experimental group comprised 66 participants (36 men and 30 women) ranging between 46 and 86 years of age, all with diabetes mellitus. In the experimental group, 21 participants suffered from atherosclerosis. All were subjected to the measurements of both the carotid-femoral pulse wave velocity (cfPWV) and the spring constant method. The comparison (control) group comprised 66 normal participants (37 men and 29 women) with an age range of 40 to 80 years who did not have diabetes mellitus. All control group members were subjected to measurement by the spring constant method.

**Results:**

Statistical analysis of the experimental and control groups indicated a significant negative correlation between the spring constant and the cfPWV (*P* < .001; *r* = - 0.824 and – 0.71). Multivariate analysis similarly indicated a close relationship. The Student’s *t* test was used to examine the difference in the spring constant parameter between the experimental and control groups. A *P-*value less than .05 confirmed that the difference between the 2 groups was statistically significant. In receiver operating characteristic curve (ROC), the Area Under Curve (AUC, = 0.85) indicates good discrimination. These findings imply that the spring constant method can effectively identify normal versus abnormal characteristics of elasticity in normal and diabetic participants.

**Conclusions:**

This study verifies the use of the spring constant method to assess arterial elasticity, and found it to be efficient and simple to use. The spring constant method should prove useful not only for improving clinical diagnoses, but also for screening diabetic patients who display early evidence of vascular disease.

## Background

Arterial elasticity is a significant predictor of cardiovascular risk. Many studies have consistently linked a decrease in arterial elasticity to both Type 1 and Type 2 diabetes [1-3]. This finding may indicate an important pathway linking diabetes to increased cardiovascular risk and mortality [4,5]. Researchers have proposed several methods of measuring arterial elasticity, including the pulse wave velocity (PWV) method, the arterial ultrasonography method, compliance, augmentation index (AIx), and the second derivative of photoplethysmogram (SDPTG). The PWV method measures the pulse propagation speed, whereas arterial ultrasonography relates the change in diameter of an artery to its distending pressure. The method of compliance, AIx, and SDPTG all evaluate arterial elasticity using arterial pressure waveforms [6-8]. Different methods are suitable for assessing arterial elasticity in different regions. The PWV method can be used to assess regional stiffness. The ultrasonography method is appropriate for assessing local stiffness, and the compliance method is suitable for assessing systemic stiffness [9]. Aortic PWV measures the speed at which a pressure wave is transmitted from the aorta to the vascular tree. The carotid-femoral PWV (cfPWV) is related to the aortic PWV and is considered the gold standard for measuring aortic stiffness. The new guidelines for the management of arterial hypertension presented by the European Society of Hypertension and European Society of Cardiology indicate that a threshold of cardio-femoral PWV exceeding 12 m/s is an indicator of subclinical organ damage [10,11]. Furthermore, the cfPWV method can also be used for the analysis of the arterial elasticity of diabetic patients [12-15]. However, all these methods are essentially indirect methods of assessing arterial elasticity, and include inconveniences in signal measurement.

Of all the methods of assessing arterial elasticity, the arterial pulse plays an important role. The pulse is generated by the left ventricle and propagates from the aorta to the peripheral arteries, such as the radial and femoral arteries. The arterial pulse is distorted from its original pulse waveform in whatever region the artery is hardened, and then propagates to the end of the artery. As a result, the peripheral arterial pulse waveform provides information on the arterial elasticity of the central or peripheral artery. Among peripheral arterial pulses, the pulse at the radial artery is the most easily and conveniently detected signal. Thus, an increasing number of studies have focused on the radial arterial pulse, with the goal of finding its time-domain and frequency-domain features to detect certain early cardiovascular-related diseases. For example, researchers have attempted to estimate the aortic artery pulse using radial tonometry by a transfer function [6,16-18].

Previous research proposes a direct measurement method of peripheral arterial elasticity. This method, which is based on the highly elastic structure of the arterial wall, uses an elastic spring to model the radial vibration of the peripheral arterial wall at the radial artery [19]. The characteristic parameter of the spring is the spring constant, which represents the ratio between exerted force and displacement according to linearity and Hooke’s law. Unlike the value of the spring constant of a normal spring, a lower value is often used to simulate the result of aging or damage to the spring in a mechanical system [20-22]. In other words, a lower value for the spring constant denotes that the elasticity of the spring has deteriorated. Similarly, a lower value for the spring constant implies deterioration in the elasticity of the arterial wall. Previous research verifies this inference [19].

Through careful verification and correlation analysis with the cfPWV, previous study confirms that the spring constant method can effectively evaluate the elasticity of the arterial wall. Because the elasticity of the arterial wall has important implications for complications such as cardiovascular-related diseases in diabetes mellitus, long-term diabetic patients require an effective and convenient index to monitor the characteristics of the arterial wall. Therefore, this study evaluates the elasticity or stiffness of the radial arterial wall in type 2 diabetic patients using both the spring constant and cfPWV methods, and compares these characteristics with those of control participants.

## Methods

### Basic theory of the spring constant method

This section briefly reviews the basic theory of the spring constant modeling. In traditional hemodynamics, the major kinetic energy of blood moves in a longitudinal direction; thus, the radial dilation is assumed to be small or negligible. The Moens-Korteweg equation and the related pulse wave velocity are based on these assumptions. Pulse wave velocity is further used as an indirect index of arterial stiffness. However, over 90 % of the energy for an in situ artery is stored in the arterial wall, and less than 10 % is stored in the circulating blood [23,24]. Thus, the vibration in a radial (transverse) direction should be emphasized over the axial (longitudinal) direction. In circulation physiology, the left ventricle ejects blood fluid into the aortic arch, which comprises a large turn, and transforms most of the axial kinetic energy into radial potential energy. Because the arterial wall (especially the media layer) consists mainly of smooth muscle cells and elastic tissue, it behaves like a spring and can transform the pumping force from the left ventricle into radial vibration of the artery. Thus, the assumptions of traditional hemodynamics should be modified, and the radial dilation should not be viewed as small or negligible. The corresponding pressure wave equation of this important concept can be derived as follows [25,26]:

(1) $$\frac{\partial^{2}P(z,t)}{\partial t^{2}}+b\frac{\partial P(z,t)}{\partial t}+\nu_{0}^{2}P(z,t)=V_{\infty }^{2}\frac{\partial^{2}P(z,t)}{\partial z^{2}}$$

Where *P* (*zt*) is the radial pressure defined as the difference between the internal fluid pressure and the fluid pressure in the static condition. The attenuation term *b* is related to the kinetic viscosity of the artery wall, the adherent fluid in the radial direction, and the stretching and contraction of the arterial wall itself. The characteristic angular frequency *v*_*o*_ is related to Young’s modulus, arterial compliance, the mass of the wall, the adherent fluid, and the radius of the tube. The symbol $V_{\infty }$denotes the high-frequency phase velocity related to the shear modulus of the wall. $V_{\infty }^{2}\frac{d^{2}P(t)}{dz^{2}}$ is the term resulting from the Windkessel effect [25].

To determine the propagation pressure wave in the peripheral artery at a fixed position, Eq. (1) is simplified to the following equation:

(2) $$\frac{d^{2}P(t)}{dt^{2}}+b\frac{dP(t)}{dt}+\nu_{0}^{2}P(t)=-k_{p}{}^{2}V_{\infty }^{2}P(t)$$

**Figure 1:**
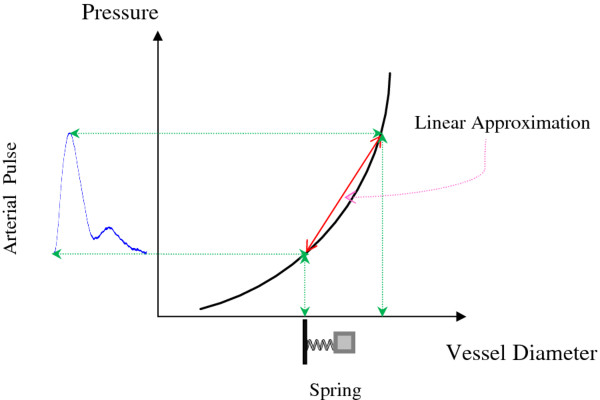
The concept of linear approximation of the pressure-diameter relationship.

Where *k*_p_ is the wave number [25]. In a relatively small range of pressure variation, the relationship between pressure and vessel diameter can be approximated to be linear; thus, *x*(*t*), the displacement of the arterial wall is also linearly related to *p*(*t*) [27,28]. Figure 1 shows the assumption of linear approximation. Consequently, Eq. (2) can be transformed into the following:

(3) $$\frac{d^{2}x(t)}{dt^{2}}+b\frac{dx(t)}{dt}+kx(t)=-k_{p}{}^{2}V_{\infty }^{2}x(t)$$

Equation (3) is analogous to describing a unit mass spring system with external force ($F_{\text{external}}=k_{p}^{2}V_{\infty }^{2}x(t)$), damping force ($b\frac{dx(t)}{dt}$), and a spring constant ($k=\nu_{0}^{2}$).

In physics, elasticity is the physical property of a material that returns to its original shape after an external deforming force is removed. The stiffness, *k'*, of a body is a measure of the resistance offered by an elastic body to deformation. For an elastic body with a single degree of freedom, stiffness is defined as k'=Fδ. The force applied to the body is denoted by *F*, and δ represents the displacement produced by the force. A spring constant can be used as a measure of the force required to achieve a particular extension. For a given displacement, *x*(*t*), the larger the value of *k*, the greater the restoring force; that is, the elastic muscle fiber of the artery wall produces greater force to restore its original shape. Accordingly, using the spring constant to assess elastic force makes it possible to evaluate the characteristics of the elastic muscle fiber. These characteristics are related to the so-called elasticity or stiffness of the arterial wall [29].

### Pulse Acquiring System

**Figure 2:**
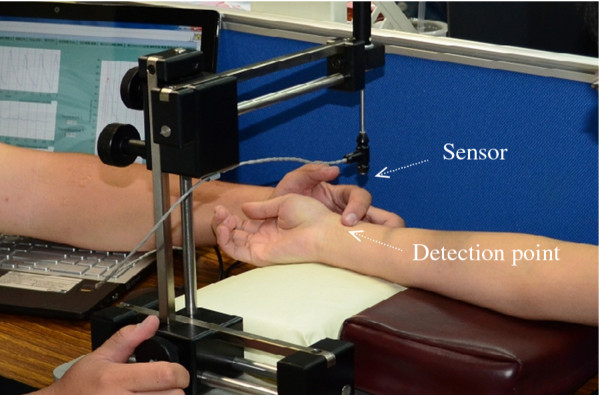
Schematic diagrams of the pulse-acquiring instrument and the detection position of the radial arterial pulse at the wrist of the right hand.

Figure 2 illustrates the proposed pulse-acquiring instrument. We measured the radial arterial pulse at the wrist of the right hand, adjacent to the ventral surface of the radial styloid process (Figure 2). The pulse recorder was used to record the radial arterial pulse. The peak and valley of the radial pulses were respectively calibrated using the SBP (Systolic Blood Pressure) and DBP (Diastolic Blood Pressure) of an oscillometric cuff system (Omron Healthcare Co., Ltd.). Measurements were taken immediately after the radial pulses in the same arm. Three supine blood pressure measurements were taken 3 min apart, and the average of the 3 readings was used.

The pressure sensor was mounted in a holder that allowed it to travel in 3 dimensions. The patient’s wrist was held motionless and adjusted in such a manner that the sensor made direct contact with the skin at the desired position on the radial artery. The measurement and analysis procedure took approximately 3–5 min when executed by an experienced operator. A pulse sequence of 10 s (roughly 10 pulses) was selected. Finally, we removed a continuous, piecewise linear trend to reduce the respiratory effect on pulse waveform. Within-operator and between-operator analyses revealed significantly high reproducibility [19].

**Figure 3:**
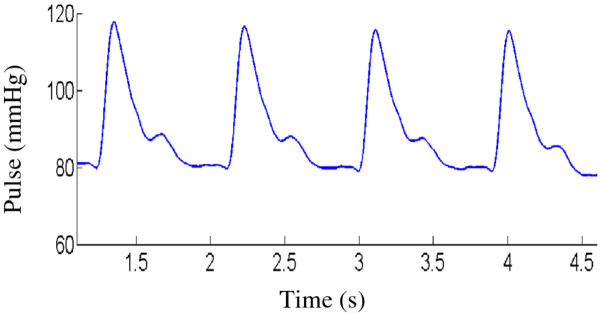
The acquired optimum pulse sequence.

To measure the arterial pulse, we gently compressed the radial artery against the bone to detect the pulse waveform of the radial artery with low distortion. Adjusting the pressure sensor vertically or horizontally obtained the maximum amplitude of the arterial pulse under various contact pressures, and then the acquired pulse was supposed to be optimum. In other words, the sensor was positioned exactly over the radial artery. With the optimum contact pressure (i.e., transmural pressure = 0), the sensor was considered well matched with the vibration of the arterial wall. Greater or lesser contact pressures would caused a distortion in the arterial pulse waveform and the spring constant [30-32]. The optimum pulse accurately reflects the vibration of the arterial wall, and can therefore be used to evaluate arterial elasticity. Figure 3 shows the acquired pulse sequence on the radial artery at the wrist. Some physiological factors, such as heart rate or respiration, may exert a small influence on arterial displacement. Thus, the arterial pulse waveforms for each pulse are not identical. Consequently, the values of the calculated spring constants for each pulse vary slightly. To reduce these influences, the analysis in this study uses the average of the spring constants of 5 steady pulse signals.

### Spring constant calculation

**Figure 4:**
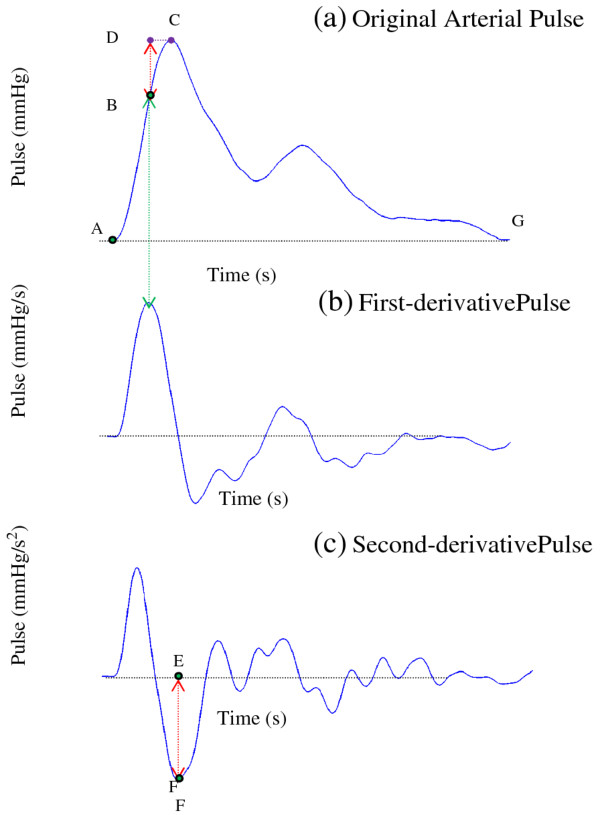
**(a) The oscillation procedure of the arterial pulse is separated by points A, B, and C, and divided into two segments A → B and B → C.(b) The first-order differential curve of the arterial pulse waveform. (c) The second-order differential curve of the arterial pulse waveform.**

The arterial pulse is separated by points A, B, C, and G, and divided into 3 segments, A → B, B → C, and C → G, as Figure 4(a) shows. The points A and C represent the valley and peak of the arterial pulse, respectively. While the arterial wall dilates from A to B, it is driven by the force because of the Windkessel effect, the restoring force of the spring, and damping force. The force caused by the Windkessel effect acts on the arterial wall mainly between points A and B, since the peak of blood flow in the peripheral artery occurs approximately within this period [33]. The total blood flow gradually decreases between points B and G, and greatly decreases the driving force generated by the Windkessel effect. At point C, the driving force caused by the Windkessel effect can be disregarded. B is defined as the maximum velocity point, that is, the equilibrium point while the arterial wall dilates from A to C [29].

Because the first derivative of the arterial pulse waveform denotes the moving velocity of the arterial wall, the maximum velocity point can be derived by finding the peak of the first-order differential curve of the arterial pulse waveform (4(b)). At point B, the second derivative of the arterial pulse waveform (Figure 4(c)) relating to the force is 0. Thus, the net (total) force acting on the artery wall is 0 according to Newton’s laws, and the arterial wall simultaneously approaches maximum velocity. Therefore, this point is considered an equivalence point in spring vibration. As the dilation extends from B to C, the restoring force of the spring and the damping forces become the major forces acting on the arterial wall. However, at the end point C of this dilation process, the velocity of arterial wall is 0, resulting from the zero value of the first derivative of the arterial pulse waveform illustrated in Figure 4(b), and starts to contract. Consequently, no damping force acts at point C because the damping force is assumed to relate to the velocity.

Therefore, at point C, the damping force and the external force originating from the Windkessel effect are both 0. Equation (3) can then be simplified as follows:

(4) $$\frac{d^{2}x(t)}{dt^{2}}|{}_{\text{at point C}}+k*x(t)|{}_{\text{at point C}}=0$$

The spring constant *k* of the arterial wall is derived as

(5) $$k=-\frac{\frac{d^{2}x(t)}{dt^{2}}|{}_{\text{at point C}}}{x(t)|{}_{\text{at point C}}}=-\frac{-\overset{―}{EF}}{\overset{―}{BD}}=\frac{\overset{―}{EF}}{\overset{―}{BD}}$$

Where *k* signifies the elasticity of the arterial wall. The symbol $\overset{―}{BD}$ represents the displacement between the equivalent point B and point C. The symbol $\overset{―}{EF}$ denotes the magnitude of the second derivative of the arterial pulse waveform at point C. Point C plays an important role in calculating the spring constant *k*.

### Pulse measurement experiment

The research parameters were measured in the study participants between November 15, 2010, and August 30, 2011, at hospital in Taiwan. The experimental protocol was approved by Institutional Review Board of Taichung Hospital, and written informed consent was obtained from all participants before they enrolled in the study. We designed an experiment to investigate the arterial elasticity in type 2 diabetic patients using both the spring constant and cfPWV methods, and then compared the results with those of the control participants. According to the World Health Organization (WHO) definition of diabetes, the inclusion criteria for type 2 diabetic patients includes a fasting plasma glucose ≥ 7.0 mmol/L or an oral glucose tolerance test (OGTT) 2-h postprandial glucose ≥ 11.1 mmol/L [34]. The subjects of diabetic group and control group were randomly selected. Participants with a history of heart failure, arrhythmia (atrial fibrillation, atrial flutter, and so on) were excluded because such diseases may affect the propagation speed of the arterial pulse. The inclusion criteria of control group included sex, age, and body mass index (BMI) that matched the diabetic patients. Subjects with a history of diabetes mellitus or atherosclerosis were excluded from the control group.

Members of the experimental group included 66 participants with diabetes mellitus (aged 46 to 86 years; 36 men and 30 women) subjected to measurement by both the cfPWV and spring constant methods. Participants in the control group of 66 normal participants (aged 40 to 80 years; 37 men and 29 women) were measured only by the spring constant method. All participants were asked not to imbibe any alcoholic or caffeinated beverages on the day of the experiment. For pulse measurement, participants were instructed to sit and relax for 5 min prior to the radial pulse measurement to reduce respiration interference. The patient’s right hand was placed on the measuring platform (Figure 3), and the operator took 10 arterial pulses at the wrist. Five steady pulse signals were selected in sequence for the spring constant calculation, and these 5 calculated values were averaged to represent the spring constant of the participants. For the cfPWV measurement, participants were instructed to rest in a supine position for 5 min, after which an automatic device (SphygmoCor system, AtCor Medical, Australia) was used to obtain the measurement.

### Statistical analysis

We used the statistical toolbox in Matlab 7.11. Data were expressed as means and standard deviations, and the Student’s *t*-test was used to compare group differences. Simple and multiple regression analyses were performed to identify the relationships between arterial elasticity measures and other hemodynamic and clinical variables. A value of *P* < .05 was considered statistically significant.

## Results

**Figure 5:**
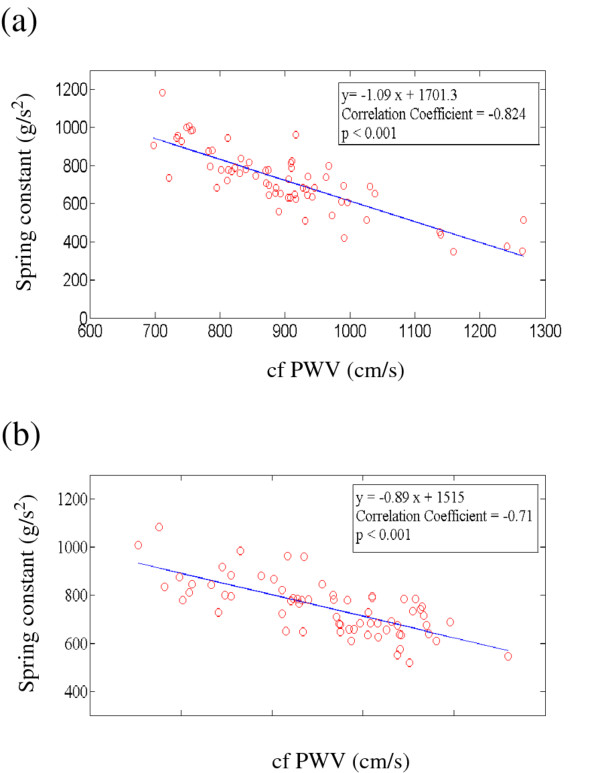
(a) Plot of the spring constant versus the cfPWV for experimental group. (b) Plot of the spring constant versus the cfPWV for control group.

Table 1 shows a summary of the means and standard deviations for the clinical characteristics of both the diabetes-patient and normal-participant groups. The radial pulse measurement was started 5 min after the cfPWV measurement. Table 1 shows the evaluated cfPWV, spring constants, and *P-*values of the experimental and control groups. Figure 5(a) plots the spring constant versus the cfPWV of the experimental group. As the cfPWV increased, the spring constant tended to decrease. This tendency is reflected by the negative-slope line (y=-1.09x+1701.3) produced by the linear regression method. The *P-*values and correlation coefficients (< .001 and -.824) indicate a significant negative correlation between the spring constant and the cfPWV. Thus, if the calculated value of the spring constant is lower, the value of the cfPWV is higher. This finding implies that the elasticity of the arterial wall deteriorates over time, possibly through aging, damage, or diabetes mellitus. The control group revealed a similar relationship, as Figure 5(b) shows. We used the Student’s *t* test to analyze the differences between the experimental and control groups for the spring constant. The resulting *P*-value (< .05) indicated a significant difference in this parameter between the two groups. This finding implies that the spring constant method can distinguish between normal and abnormal elasticity characteristics in normal and diabetes participants as effectively as the cfPWV [12-15].

**Table 1:** **Clinical characteristics of the diabetic patient group, normal subject group, and** ***P-*** **value**

	Diabetic patient group		Normal subject group		*P-*value
Variable	Mean	Standard Deviation	Mean	Standard Deviation	
Age (years)	65.1	11.6	62.3	9.5	.15
Weight (kg)	69.5	11.8	66.7	10.6	.12
Height (cm)	164.6	6.4	163.5	9.8	.53
BMI (kg/m^2^)	25.5	2.1	24.9	2.1	.36
Brachial SBP (mmHg)	121.2	8.7	118.7	13.8	.08
Brachial DBP (mmHg)	78.6	4.6	74.1	10.6	.21
Heart Rate (bpm)	66.7	7.3	69.3	10.3	.09
cfPWV (cm/s)	900.3	128.2	859.1	91.5	< .05
Spring Constant (g/s^2^)	723.5	169.0	749.3	114.3	< .05

Table 2 shows the multivariate regression analysis of the relationship between the spring constant and age, height, brachial systolic blood pressure, heart rate, and cfPWV, respectively. The spring constant is independently influenced by all 5 clinical parameters, with an adjusted R^2^ of 0.59. The measure β (standardized coefficient) indicates how strongly each predictor variable influences the criterion variable. These results also imply that the spring constant is significantly related to the cfPWV (*P* < .001). The results in Table 2 show that as age, brachial SBP, and cfPWV increased, the spring constant significantly decreased, indicating deteriorating elasticity in the artery. These results agree with a basic physiological understanding and prior research findings on vessel elasticity, and confirm the validity of using a spring constant to model vessel elasticity [35,36].

**Table 2:** Multivariate relationship between spring constant and clinical characteristics of the diabetic patient group

Variable	β (Standardized Coefficient)	*P*-value
Age (years)	- 0.512	< .001
Height (cm)	- 0.315	Not Significant
Brachial SBP (mmHg)	- 0.522	< .001
Heart Rate (bpm)	- 0.335	Not Significant
cfPWV (cm/s)	- 0.725	< .001

**Figure 6:**
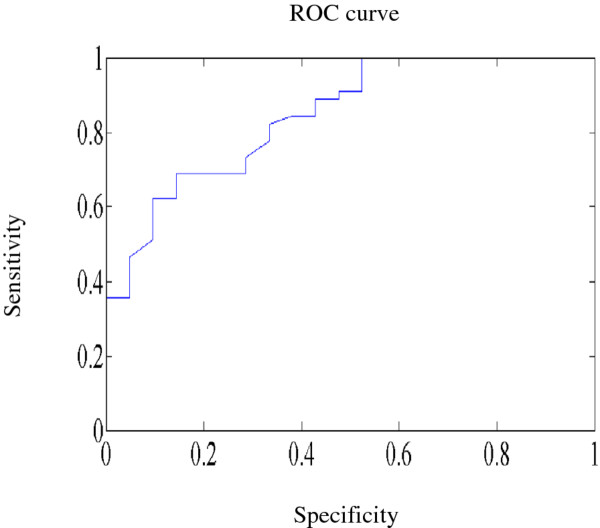
The receiver operating characteristic curve.

In the experimental group of 66 participants with diabetes mellitus, 21 participants suffered from atherosclerosis; thus, they were viewed as patients with arterial stiffness. We used the receiver operating characteristic curve (ROC) to assess the sensitivity of the proposed method to evaluate arterial stiffness (Figure 6). The Area Under Curve (AUC) (= 0.85) indicates good discrimination. The mean and standard deviation of the 21 participants with atherosclerosis are 578.8 ± 144.1 (g/s^2^). This mean value approaches the best operating point of the ROC curve. Thus, a spring constant less than the mean (578.8 (g/s^2^)) may preliminarily predict a risk of arterial stiffness for diabetic patients.

## Discussion

### Effect of diabetes on arterial elasticity

The possible reasons for increased arterial stiffening in type 2 diabetes include impaired glycemic control and the formation of advanced glycation endproducts (AGEs) that destroy the function of endothelial cells and lead to structural changes in the vessel walls [37,38]. Arterial stiffness is related to endothelial dysfunction, which leads to an imbalance in the release of vasoactive substances from the endothelium. Smaller arterioles and branch vessels are more prone to be affected than larger arteries because the thinner media of the smaller arteries responds strongly to the endothelial release of nitric oxide. Thus, the presymptomatic monitoring of the arterial elasticity of both large and small arteries in patients with type 2 diabetes should decrease the risk of cardiovascular complications. Many studies have demonstrated an increased arterial stiffening of the large artery in both type 1 and type 2 diabetes using both central PWV and central pulse pressure [1,39]. The stiffening of the peripheral artery may be more obvious than that of the larger central arteries in type 2 diabetic patients [40]. Nevertheless, few studies have discussed the effect of diabetes on the elasticity of small or peripheral arteries.

### Validity of spring constant method

Some noninvasive modalities, such as MR imaging (MRI), computed tomography (CT), and digital subtraction angiography (DSA) can provide more accurate information about plaque, stenosis, or stroke [41]. In addition, the circulating level of microparticles, oxidized low-density lipoproteins, and intima-media thickness are associated with the arterial elasticity of diabetes patient [42-44]. However, these techniques are expensive and inconvenient, and are therefore not suitable for the early and fast diagnosis of arterial stiffness.

Because the radial pulse is easily detected at the peripheral artery, it is feasible to quickly evaluate the elasticity of the peripheral artery of diabetic participant using the radial pulse waveform. Using the proposed spring constant method, this study analyzes the peripheral arterial elasticity of diabetes and normal participants. Results show that the spring constants of diabetes participants are significantly lower than those of normal participants. This implies that the arterial stiffening of diabetes participants is more serious than that of normal participants. These results agree with prior research. This study also evaluates the sensitivity of this method based on the receiver operating characteristic curve, and the value of AUC shows good discrimination. Although more subjects are required to define the risk value of spring constant for arterial stiffness, a spring constant below 578.8 (g/s^2^) may imply a risk of arterial stiffness for diabetic patients in this study. This suggests that the spring constant method can effectively distinguish between peripheral arterial elasticity in the experimental and control groups. In addition, the spring constants of diabetic participants and normal subjects are significantly correlated with their cfPWV values. This confirms the accuracy of the spring constant method for assessing peripheral arterial elasticity.

The pressure-diameter or stress–strain relationship of the artery wall is non-linear when pressure and diameter are observed over a wide range [27]. However, the dynamic variation in arterial pulse pressure is relatively small compared with the overall blood pressure. Thus, a linear approximation of the pressure-diameter or stress–strain relationship is feasible. Because of the complexity of a non-linear physiological system, the assumption of linearity is an essential approach to find the average effect or tendency of a complex system. Although approximations may cause some distortion, the significant correlation between the cfPWV and the spring constant in these results confirms the feasibility of a linear approximation.

Currently available methods are basically qualitative and indirect in principle. For example, some problems still occur with PWV. First, arterial stiffness does not affect PWV independently. The contraction ability of the left ventricle, the blood viscosity, and the resistance of the terminal vascular bed may also affect PWV. Second, it is difficult to estimate the actual wave travel distances [45,46]. In contrast, the spring constant method directly and quantitatively evaluates peripheral arterial elasticity. It is theoretically based on the pressure wave equation, and can simply derive the spring constant using 1 optimum pulse at a single measurement position. The proposed method requires only a few minutes to measure a radial pressure pulse and calculate the spring constant, making it convenient and practical for clinical use.

Although the radial pulse is detected at the radial artery, it propagates from the heart, through the aortic region, and to the peripheral artery. Thus, this pulse measurement may include information about systemic, regional, or local arterial stiffness, and it can be used to assess arterial elasticity [10,47]. Stiffened arteries may cause sudden death from events such as stroke or myocardial infarction, which often occur in diabetic patients. Thus, effective long-term monitoring of the status of arterial elasticity is an important issue for diabetic patients. The method proposed in this study has the advantage of directly measuring peripheral arterial elasticity. The spring constant method should prove to be useful not only for improving clinical diagnosis, but also for screening diabetic patients for early signs of vascular disease.

## Abbreviations

PWV = pulse wave velocity; cfPWV = carotid-femoral pulse wave velocity; AIx = Augmentation index; SDPTG = Second derivative of photoplethysmogram; AGEs = Advanced glycation endproducts.

## Competing interests

The authors declare that they have no competing interests.

## Authors’ contributions

C-C W carried out the research design, performed the statistical analysis, and prepared the final manuscript. S-W H participated in the design of this study and result interpretation. C-T B made contributions to the acquisition of data. All authors read and approved the final manuscript.
